# Validation of the stratify scale for the prediction of falls among hospitalized adults in a tertiary hospital in Colombia: a retrospective cohort study

**DOI:** 10.1038/s41598-023-48330-y

**Published:** 2023-12-07

**Authors:** Olga L. Cortés, Skarlet Marcell Vásquez, Angie Cristina Mendoza

**Affiliations:** 1 Research Unit and Nursing Department, Fundación Cardio Infantil Instituto de Cardiología, Cl. 163a #13B-60, Bogotá D.C, Colombia; 2 Faculty of Nursing, Universidad Autónoma de Bucaramanga, Avenida 42 No 48-11PBX, Bucaramanga, Colombia; 3 Fundación Cardiovascular de Colombia, Calle 155A No. 23 - 58, Floridablanca, Colombia

**Keywords:** Health care, Risk factors

## Abstract

The STRATIFY scale has been implemented as a preventive strategy for predicting the risk of accidental falls among hospitalized adults. However, there is still uncertainty about its accuracy. This study aimed to perform an external validation of the STRATIFY fall prediction scale in hospitalized adults in one tertiary care hospital in Bogotá, Colombia. The study was a retrospective cohort of adult hospitalized patients in a high-level complexity care hospital. The sample selected included admitted patients (age ≥ 18), consecutively by the institution between 2018 and 2020, with an evaluation of the fall risk measured by the STRATIFY score given to each at the time of hospital admission. For assessing the scale's feasibility, its discriminative capability was obtained by calculating sensitivity, specificity, likelihood ratios, predictive values, and area under the ROC curve. The evaluation included 93,347 patient hospital records (mean 56.9 years, 50.2% women). The overall sensitivity score was 0.672 [IC 95% 0.612–0.723], the specificity score was 0.612 [IC 95% 0.605–0.615], and the positive likelihood ratio was 1.73 [IC 95% 1.589–1.891]. The area under the ROC curve was 0.69 [IC 95% 0.66–0.72]. Subgroups of age obtained similar results. Applying the STRATIFY scale at hospital admission resulted in a lower performance of the tool–predict falls in hospitalized patients. It is necessary to implement an individual evaluation of the risk factors for falls in order to structure appropriate care plans to prevent and improve hospital safety.

## Introduction

Falls incidence in adult-hospitalized patients is between 1 and 25%, depending on the hospitalization service^1–5^. It is estimated that more than 84% of the adverse events in hospitalized patients are related to accidental falls, placing this event as the first and most notified in the year 2021 in the sentinel event database of the Joint Commission International^6^.

For the most part, falls generate minor lesions. However, a tenth of these events result in more severe patient lesions, such as concussions, fractures, or death, impacting not only health-related but also social and economic aspects^7–9^. The World Health Organization (WHO) considers them adverse events that all health systems must control. Falls make up 37% of deaths from lesions and more than 34,000 hospitalizations (3.2 for every 1000 hospitalizations). Moreover, the total cost of a fall on hospital grounds is around $9.8 million, of which $6.4 million pertains to falls without lesions and $3.4 million to falls with lesions^1–6^. Some of the consistent factors associated with the risk of falls are advanced age, intake of sedatives, mental and behavioral disorders, cardiovascular-derived factors (syncope, hyperkalemia, hypotension), and those which limit mobility in patients: balance alteration and aid requirement for walking^10–14^.

Identifying vulnerable patients with a high risk of falls could aid in reducing the incidence of this event when implementing specific strategies for prevention. Until now, an array of different instruments has been developed for measuring the prediction of the risk of falls^15^. However, it has yet to be identified that any of these have a superior discriminatory capacity compared to the others or that it is better than a clinical judgment of fall risk assessment^15,16^. Some nursing clinical practice guidelines^17–19^ advise that according to the available evidence, the use of prediction tools as the unique strategy for hospital screening is unfavorable and suggest structuring other ways for evaluating the risk of falls given the multi-morbidity of most hospitalized patients^17–19^.

Despite these recommendations, these instruments are still widely used in clinical practice due to their low application complexity and some Guidelines that make them essential^6^. However, the capability limitations of these tools' prediction could generate an inadequate identification for these patients, leaving some at a higher risk of falling and even more exposed to this event.

The scale that is mainly applied compared to the other tools for determining the risk of falls is the STRATIFY clinical scale for fall prediction (St. Thomas Risk Assessment Tool in Falling Elderly Inpatients). This tool includes five predictors of risk: History of falls, patient agitation, visual impairment, incontinence, transference, and mobility. The STRATIFY score is the sum of the presence or absence of each predictor of risk, varying from 0 to 5 points [a score of < 2 equals a low risk of falls, and a score ≥ 2 equals a high risk of falls] being the "cut-offs" a score ≥ 2^15,20^.

Although the STRATIFY scale has been evaluated worldwide since the first validation in 1997^20^ its discrimination capacity remains ambiguous to be used with patients at high risk. Its sensibility is from regular, around 80% to very low around 65% and ≤ 65%, as well as its specificity that is around 71–51.2%^21–23^; and it has a relatively high negative predictive value (NPV) [86.5 95% CI 78.4, 94.6] and a low positive predictive value (PPV) [23.1 95% CI 43.0, 59.3] that may not be optimal for identifying high-risk individuals for fall prevention^21–23^. These results are similar to those reported in other studies (Matarese^22^; Strini) in which a low sensitivity of the scale in the young population is also evident^22^.

The STRATIFY scale was implemented for preventive care in our hospital in 2010 in patients above 18 years old, given that the mission of this hospital is to give care to patients under high and very high cardiovascular risk with high quality of care. Thus, this tool was implemented in our hospital to identify patients at risk of falling. All patients are assessed at hospital admission and at least once every 24–48 h during their hospital stay. In the emergency room and hospitalization facilities, the nursing staff is responsible for assessing the patient's risk by administering the Stratify scale. The hospitalization rate of patient falls in the Fundación Cardioinfantil Instituto de Cardiología-La Cardio [FCI-IC] was 0.69 per thousand hospitalized patients. The incidence rate of patient falls in the Emergency Department was 0.64 per thousand patients treated in 2019 [Below the incidence of 0.85 in hospitalization and 1.05 in emergencies reported for Bogotá, and 1.03 in hospitalization and 0.99 in emergencies reported for Colombia]^24^.

Even though this predictive tool has been implemented for many years along with other prevention strategies for preventing falls, its incidence is still elevated.

Thus, before implementing any other plan oriented to the reduction of the incidence rate of falls, we decided to perform an external validation of the STRATIFY fall prediction precision scale in hospitalized adults in the FCI-IC, a hospital of high-complexity in Bogotá (Colombia).

## Methods

This study is reported according to the STROBE Guidelines/methodology (https://www.strobe-statement.org/checklists/).

### Study design

This study was an external validation of the predictive precision capability of the STRATIFY risk fall scale. It was conducted in a retrospective cohort study based on information obtained of clinical charts of patients admitted to one high-complexity care hospital in Bogotá (Colombia), identifying those individuals with and without falls reported during hospitalization. The study was approved by the Institutional Ethics Committee (CEIC 2020).

### Setting

The study was conducted in a reference hospital of high complexity care patients (260 adult-beds, 1200–1500 approx. patients' admittance by month). This hospital offers care wards in all medical specialties necessary for adult care. This study was developed within the scope of hospitalization services (medical and surgical) and emergency services for the adult population. The patient's admission spectrum included cardiovascular surgery, neurosurgery, orthopedic, transplants, non-cardiac surgery, and internal medicine. The STRATIFY scale is part of conventional care used routinely since 2010 by nursing staff in all patients above 18 years old once they are admitted into the hospital and at least once every 24–48 h during their hospital stay.

### Participants

Medical records of patients ≥ 18 years old and admitted into the institution between January 1, 2018, and June 3, 2020, were consecutively included as part of the sample participants. These medical records had to include the report of the fall risk assessment recorded at the time of hospital admission using the STRATIFY scale (score between 0 and 5). Independently, we did request the list of all patients who had suffered a fall during the same study period registered to the quality office of the hospital. This final registry was crossed with the list of all patients admitted into the study. Patients with duplicate records of the STRATIFY scale were excluded at admission.

### Sample size

The sample size calculation included at least 26,488 patients, following the methodology proposed by Obuchowski and McClish^25^. The calculation assumed an area under the ROC curve of 0.7, a rate of falls of 0.69 per thousand patients, a significance level of 5%, and a power of 80%.

### Data collection and procedure

Patient information was obtained with an extracting tool of digital data used by the hospital's technical systems group. The following were the variables of interest extracted from the admission records: patient ID, age, clinical diagnosis, and STRATIFY score at admission [registered with the same date]. Moreover, the following variables of interest were extracted from reports of clinical incidents from the hospital quality control office: ID from patients who reported a fall event, date of the event, place, and time.

Once the variables were obtained, information about patient records and reported falls were crossed with admission records. Then we built a database with all the described information and finally identified those who suffered a fall and those who did not (binary data), with each of their pertaining scores for the falls risk assessment tool STRATIFY (0–5). Afterward, patients were classified accordingly at low risk or high risk of falls, depending on the conventional cut-off point of the scale: < 2 points for low risk (negative) and ≥ 2 for high risk (positive).

### Definitions

True positive rate: Patients who fell with a high-risk rating (≥ 2).

True negative rate: Patients who did not fall and had a low-risk rating (< 2).

Predictive positive value: The probability of fall having high risk score.

Negative predictive value: The probability to do not fall having low risk score.

LR + is equivalent to the probability that a person that falls tested positive for the risk of fall (true positive) divided by the probability that a person without falling tested positive for the risk of fall (false positive), [Sensitivity/1 − Specificity].

LR- is equivalent to the probability that a person who falls tests a negative score (false negative) divided by the probability that a person without the fall tests negative for the fall (true negative) [1 − Specificity/Specificity].

### Outcomes

The primary outcome was to determine the predictive characteristics of the STRATIFY scale: Sensibility (the proportion of people with a confirmed positive result among those who fell -with the target condition); and Specificity (the proportion of people with a negative test result among those who do not fall or without the target condition), and Likelihood Ratios (LR + , LR − , 95% CI). The secondary outcomes were the positive and negative predictive values (PV), and lastly, the area under the ROC curve was estimated (AUC).

### Statistical analysis

The descriptive analysis of the sociodemographic and clinical characteristics of the sample, were analyzed considering absolute and relative frequency measurements for discrete variables and mean (SD) for continuous variables. Also, it was described the general proportion of falls and the time and setting of the fall in hospitalization. Differences between the group of patients with falls and those without falls were evaluated using the Chi2 test and nonparametric tests (for discrete and continuous variables, respectively). The significance level for these tests was 0.05. It was structured a binary (2X2) table with the data obtained to evaluate true and false positives, false and true negatives to calculate Sensitivity, Specificity [95% confidence intervals], the Likelihood Ratios (LR + , LR − ), and the positive and negative predictive values.

Sensibility, Specificity, and other estimates were calculated according to dichotomized scores [< 2 and ≥ 2] and for the continuous scores [0 -5]. Finally, the area under the ROC curve (ROC-Receiver operating characteristic curves) was determined to provide a global representation of diagnostic accuracy, with the data obtained on Sensitivity and Specificity at the different cut-off points of the scale (0–5). All analyses were performed in subgroups of patients older than 55 and 65 years to assess the discriminating ability of STRATIFY in both groups. The analyzes were performed in Stata version 12.0.

### Ethics approval and consent to participate

This project complies with the ethical regulations mentioned in Colombian Resolution number 008430 of 1993. It is within the suggested guidelines in the Helsinki declaration of the World Medical Association, the Council for International Organizations of Medical Sciences (CIOMS), and the Belmont report. This project was approved by the ethics committee of the Fundación Cardioinfantil (IRB00007736, 044-2020, Nov 19, 2020), in the investigation category with a non-risk given it was a retrospective design. Informed consent was waived by the ethics committee of the Fundación Cardioinfantil due to the retrospective nature of the study.

## Results

Finally, 93,347 patient records were obtained and assessed for fall risk with the STRATIFY scale at the time of admission. Table 1 shows the characteristics of the included patients. The mean age was 56.9 (SD 18.5) years, and the proportion of women was 50.2%. The leading causes of hospital admission were related to cardiovascular care (22.0%), general medicine (23.3%), gastrointestinal (13.4%), and haemato-oncology (8.4%).

**Table 1:** Sociodemographic and diagnosis characteristics of patients according to fall events in the hospital.

Characteristics	Total *n* = 93,347 (100%)	Fall *n* = 250 (0.3%)	No-Fall *n* = 93,097 (99.7%)	*p-*value
Male sex, n (%)	46,507 (49.8)	142 (56.8)	46,365 (49.8)	0.87
Age years, mean (SD)	56.97 (18.5)	65.75 (18.3)	57.0 (18.5)	0.27
Age cathegory, n (%)				
≥ 18–54	39,992(41.8)	57 (22.8)	38,992 (41.8)	< 0.001
55–64	18,993 (20.4)	41 (16.4)	18,993 (20.4)	
≥ 65	35,112 (37.7)	152 (60.8)	35,112(37.1)	
System altered don admission, n (%)				
Cardiovascular	20,426 (22.0)	45 (18.0)	20,417 (22.0)	< 0.001
Neurological	5085 (5.4)	34 (13.6)	5051 (5.4)	
Gastrointestinal	12,533 (13.4)	31 (12.4)	12,502 (13.4)	
Hemato-oncology	7969 (8.4)	29 (11.6)	7840 (8.4)	
Respiratory	5824 (6.2)	18 (7.2)	5806 (6.2)	
Urological	4228 (4.5)	18 (7.2)	4210 (4.5)	
Musculoskeletal	5444 (5.8)	16 (6.4)	5428 (5.8)	
General	21,787 (23,3)	14 (5.6)	21,773(23.4)	
Infection	2699 (2.9)	14 (5.6)	2685 (2.9)	
Metabolic	1258 (1.3)	9 (3.6)	1249 (1.3)	
General surgery	1069 (1.1)	7 (2.8)	1062 (1.1)	
Transplant	1153 (1.2)	5 (2.0)	1148 (1.2)	
Circulatory	1603 (1.7)	4 (1.6)	1599 (1.7)	
Covid-19	1450 (1.6)	3 (1.2)	1447 (1.6)	
Psychiatric	326 (0.3)	3 (1.2)	326 (0.3)	
Cardiovascular surgery	557 (0.6)	0	557 (0.6)	

### Description of falls

Out of the included patients, 250 (0.3%) suffered falls, the average age was 65.7 years (SD 18.3), and 56.8% were male. The prevalence of falls by age group was 0.15% (n = 57) in patients aged ≥ 18–54 years, 0.22% (n = 42) in the group aged 55–64 years, and 0.43% in the group aged ≥ 65 years (n = 152).

Falls occurred at a higher frequency in the bathroom from the patients' same height, from the bed, and in the hospital bedroom (30.4%, 26.0%, 11.6%, and 10.0%, respectively). The highest frequency of falls was observed during the night shift (44.4%, n = 111). Twenty-four percent (n = 61) of the people who fell suffered some lesion due to the fall. Diagnosis at the time of hospital admission was primarily due to the following concerns: cardiovascular (18%), neurological (13.6%), and gastrointestinal (13.6%) (Table 1).

### Total score and risk of falls

From the total of included patients, it was identified that 36,257(38.8%) patients were identified as at high risk of falls (score ≥ 2), and 57,090 (61.2%) of them were at low risk of falls (score < 2). Of the high-risk patients, 36,089 (99.5%) were identified as false positives, and 168 (0.46%) were confirmed true positive cases. Moreover, 82 (0.14%) were considered false negatives of the total of patients at low risk, and 57,008 (99.8%) were true negatives (Table 2).

**Table 2:** Overall diagnostic characteristics of a STRATIFY in screening high risk patients of fall.

STRATIFY dichotomic score	Falls				Total
	Present	*n*	Absent	*n*	
Positive: high score ≥ 2	True positive^a^	168	False positive^b^	36,089	36,257
Negative: low score < 2	False negative^c^	82	True negative^d^	57,008	57,090
Total		250		93,097	93,347

a-True positive, b-false positive, c-False negative, d-True negative.

**Figure 1:**
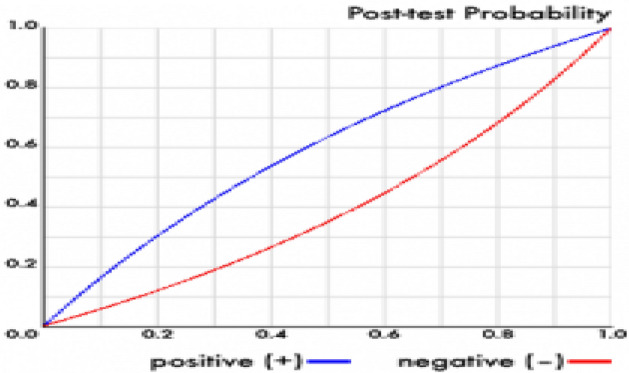
Post-test of probability.

The efficiency evaluation of the STRATIFY Scale according to the fall risk dichotomic classification (High or Low) globally showed a Sensitivity of 0.672 [IC 95% 0.612–0.723]; and a Specificity of 0.612 [IC 95% 0.605–0.615]. The Positive Likelihood Ratio (LR +) was 1.73 [IC 95% 1.589–1.891] and the Negative Likelihood Ratio (LR − ) was 0.536 [IC 95% 0.449–0.64]. The positive predictive value was 0.005 [IC 95% 0.004–0.005] and the negative predictive value was of 0.999 [IC 95% 0.998–0.999]. The general precision score was 61.25% [IC 95% 60.94%–61.57%] (Fig. 1).

The efficiency evaluation by subgroups of age are presented in Table 3, and the evaluation of sensitivity and specificity by age groups can be seen in Table 4. The results show a very low sensitivity in young patients’ group of age (≥ 18–54 and 55–64 years) and moderate in older adult patients (≥ 65).

**Table 3:** Relationship between the predictive diagnostic of risk of fall and actual presence of fall by age subgroups.

Age subgrupos	STRATIFY score	Fall	
		Present	Absent
18–54	≥ 2	26^**a**^	9758^**b**^
	< 2	31^**c**^	29,234^**d**^
Total		57	38,992
55–64	≥ 2	23^**a**^	6782^**b**^
	< 2	18^**c**^	12,211^d^
Total		41	18,993
≥ 64	≥ 2	119^**a**^	19,549^**b**^
	< 2	33^**c**^	15,563^**d**^
Total		152	35,112

a- True positive, b- false positive, c -False negative, d- True positive.

**Table 4:** The predictive diagnosis of risk of fall by age groups [cut of point ≥ 2].

Age group	Sensitivity	Especificity	Positive likelihood ratio	Negative likelihood ratio
	(95% CI)	(95% CI 95)	(95% CI)	(95% CI)
**≥ 18–54**	45.6 [32.3, 59.3]	74.9 [74.5, 75.4]	1.82 [1.37,2.42]	0.73 [0.57, 0.92]
**55–64**	56.1 [39.7, 71.5]	64.2 [63.6, 64.9]	1.57 [1.2, 2.06]	0.68[0.48, 0.97]
**≥ 65**	78.2 [70.8, 84.56]	44.3 [43.8, 44.8]	1.41 [1.29,1.53]	0.49 [0.36, 0.66]

Significant values are in [bold].

The results show a very low sensitivity in young patients’ group of age (≥ 18–54 and 55–64 years) and moderate in older adult patients (≥ 65).

The Sensitivity and Specificity for each cut-off point showed a high Sensitivity of 100% and 84% for the score of cero (0) and one (1) [if a patient has low risk]. The cut of points 2, 3, 4, and 5 showed a decreasing sensitivity of 67.2%, 30.8%, 7.6%, and 1.6% [High risk], respectively. At the cut-off point of 2, the sensitivity was 0.672, and the Specificity was 0.612 (Table 5).

**Table 5:** The trade-off between sensitivity and specificity when using all different STRATIFY scores.

STRATFY score	No fall *n* = 93,097	Fall *n* = 250	Sensitivity	Especificity	Classified	LR +	LR-
	*n* (%)	*n* (%)	(%)	(%)	(%)		
0	40,135 (43.11)	40 (16.0)	100.00	0.00	0.27	1.0000	
1	16,873 (18.12)	42 (16.8)	84.00	43.11	43.22	1.4766	0.3711
2	27,929 (30.0)	91 (36.4)	67.20	61.24	61.25	1.7335	0.5356
3	6975 (7.49)	58 (23.2)	30.80	91.23	91.07	3.5140	0.7585
4	1002 (1.08)	15 (6.0)	7.60	98.73	98.48	5.9708	0.9359
5	187 (0.20)	4(1.6)	1.60	99.80	99.54	8.1395	0.9859

**Figure 2:**
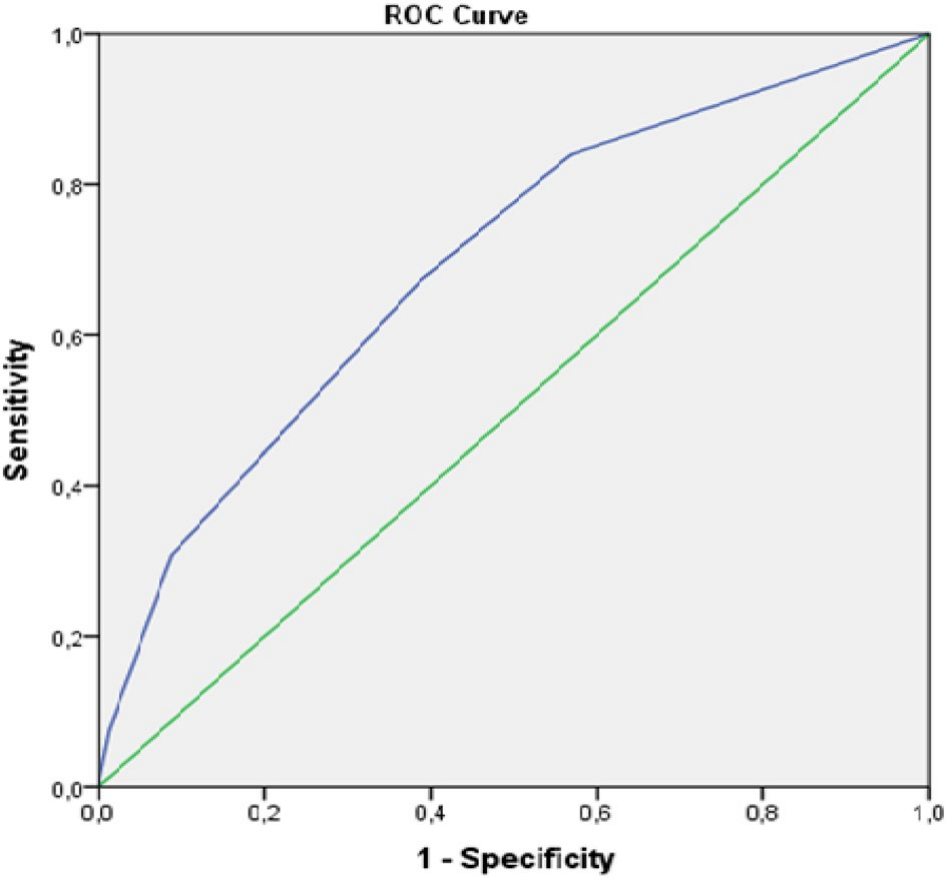
Receiver operator characteristic (ROC) curve of STRATIFY scale.

The global area under the curve including all patients was 0.695 [CI 95% 0.66–0.72] (Fig. 1). The evaluation of the area under the ROC curve for patients older than 55 and older than 65 years was very similar to the general observation (Fig. 2). For patients older than 55 (n = 52,548) the area under the ROC curve was 0.695 [CI 95% 0.664–0.727]; whilst for patients who were older than 65 (n = 33,347) the area under the curve was 0.663 [CI 95% 0.620–0.705].

## Discussion

The current study showed the very low usefulness of the STRATIFY scale in predicting falls in adults and older adults evaluated during admission to a high-complexity hospital. The results reported a regular sensitivity (67%) and Specificity (61%) at the conventional cut-off point (> 2) used in our hospital, with both positive and negative likelihood ratios of very little relevance and clinical utility in the prediction of In-hospital falls [LR positive < 0.2 and negative LR > 0.5]. The overall LR (1.73) showed a small and rarely important change from pretest to post test probabilities (from 30 to 40%), that was also similar observed by age subgroups. The changes do not make the test clinically useful^26,27^.

Screening tools for patients at risk of falls are a helpful complement due to their predictive capacity, capable of establishing this diagnosis in the hospitalized adult population. Once the type of risk and the individual factors that can cause a fall are established, it becomes possible to organize a preventive care plan. Advantageous scales must show an excellent capacity for risk stratification and prediction of falls (greater than or equal to 95%). That was the initial purpose of the original study that validated the STRATIFY scale developed by Oliver^21^. This study presented an excellent sensitivity of 93% and a moderate specificity of 88%, obtained in validating the instrument in the geriatric population. However, the risk discrimination capacity of the STRATIFY scale in patients with different ages and pathologies and hospitalized in different types of services presents significant variability and disagreement^13,21,23^. The differences between the risk and the presence of falls observed in the evaluated patients lead to damage or injury in hospitalized patients with severe implications for hospital safety, quality of life [for the patient and his family], and tremendous implications for the General Health System^1^. Our study showed a low sensitivity to identify patients at risk of falling at all cut-off points of the scale above 2, given a low positive predictive value and a very high negative predictive value.

Among the studies with results similar to ours is the systematic review and meta-analysis carried out by Billington et al.^28^, which included 17 validation studies of the STRATIFY scale in a total population of 11,878 adults from different contexts (hospitals, geriatric institutions, or nursing homes). The overall estimate for sensitivity was 67%, and specificity 57%. The Sensibility and Specificity reported for fall prevalence of less than 10% increased to 75% and 63%, respectively. In this case, these subgroup estimates' 95% confidence intervals were more extensive than the overall estimate, reflecting limited diagnostic precision^28^.

These findings remain consistent in patients of different ages and contexts with a low prevalence of falls. For example, Castellini et al. published 2017 a retrospective observational study to validate the STRATIFY falls prediction scale in patients older than 75 years, hospitalized in neurology units in Italy, who were evaluated with this tool on admission. A rate of 0.9 falls per 1000 patient days was obtained, and the reported sensitivity and Specificity for the scale were 35.6% and 64.4%, respectively^29^. Similarly, a prospective cohort study published in the same year, conducted in five hospitals in Spain, included 1247 patients older than 16 years hospitalized in intensive care and reported an incidence of falls of 2.35%. The study results showed a sensitivity of 47.5% and a specificity of 85% (for the cut-off point ≥ 2 points, the area under the ROC curve of 0.69 [95% CI 0.57–0.8])^30^.

Difficulties in screening high-risk patients have been reported in several clinical practice guidelines. These observations are mainly related to high variability in the estimate observed between different scales^17–19^. Based on that, the NICE Guideline (G161), in its results of updating fall risk screening tools, showed that most of the studies evaluated (including those related to the STRATIFY scale) could not screen patients at risk of falls adequately in hospitalization^18^. One explanation for this finding is related to the possibility that screening tools do not necessarily have high accuracy in populations with a low prevalence of the event, as is the case of In-hospital falls in our center. Another explanation may be related to the high variability of hospitalized patients that limits the capacity of the scale, such as the age and types of diagnoses of each patient who is hospitalized in different services or care units and multiple diversity of complications^13^. In other words, the lack of precision of the STRATIFY scale in hospitalization may be determined by the presence of multiple factors that are determinants of the health status of highly compromised patients, which can be determinants of a fall but need to be contemplated on the scale.

Considering our results, we appraise that there is no certainty for using the STRATIFY scale in hospitals like ours, which admit highly complex patients who must be provided with high security of care. We propose to follow the NICE guidelines, which recommend, before using a fall risk scale, the performance of an initial assessment of the patient's status, recognizing the possible factors individually that can lead to a fall and thus establishing proper care plans oriented toward reducing the risk of falls.

### Strengths and limitations

The present study has several strengths. It is one of the first studies conducted in our country, including a large sample size of 93,347 patients, to validate the discrimination capacity of the STRATIFY scale. The large sample size of patients provided validity to the overall and age subgroups of patients of the diagnostic characteristics of STRATIFY in various care services of different complexity. These results provided advantageous knowledge to guide the decision-making about using this tool to discriminate patients at risk in our hospitals, health care providers, and the decision makers of the health system in our country. Furthermore, our results apply to hospitals that treat similar adult patients evaluated at admission time. The results may help other hospitals decide whether to implement or revalidate it according to the patient's risk.

Our hospital counts on a fall prevention group and a program for reliable reporting of events (by nursing staff, family members, or self-reporting of the event by patients), which increases the sources of information about the event. Given that nurses and auxiliary caregivers provide this tool to patients, this could be a source of bias affecting the accuracy of some patients' scores^31,32^.

### Implications for clinical practice

Care should be taken in extrapolating disease prevalence from diagnostic accuracy studies in other populations. Likewise, it is essential to keep in mind that patients, after the moment of admission and according to their health status and complications associated with the progress of the disease and type of treatment, can modify their risk score. For this reason, it would be much more reasonable to evaluate individualized risks to structure care plans that prevent said risks, which can be modified during the hospital stay^32^.

## Conclusions

Applying the STRATIFY scale in hospital admission presented a low performance in predicting falls in hospitalized adult patients. Fall risk assessment using the STRATIFY scale in this population is not recommended as a safe tool for fall prevention.

## Data Availability

All the data supporting the study findings are within the manuscript. Additional detailed information and raw data are available from the corresponding author or Institution [responsible of the data] on reasonable request.

## Abbreviations

AUC: Area under the curve
CI: Confidence interval
ROC: Receiver operating characteristic curves
SD: Standard deviation
STRATIFY: St. Thomas Risk Assessment Tool in Falling Elderly Inpatients
